# Urolithins: The Gut Based Polyphenol Metabolites of Ellagitannins in Cancer Prevention, a Review

**DOI:** 10.3389/fnut.2021.647582

**Published:** 2021-06-07

**Authors:** Sami A. Al-Harbi, Abdulrasheed O. Abdulrahman, Mazin A. Zamzami, Mohammad Imran Khan

**Affiliations:** ^1^Department of Chemistry, University College in Al-Jamoum, Umm Al-Qura University, Makkah, Saudi Arabia; ^2^Department of Biochemistry, Faculty of Science, King Abdulaziz University, Jeddah, Saudi Arabia; ^3^Cancer Metabolism and Epigenetic Unit, Faculty of Science, King Abdulaziz University, Jeddah, Saudi Arabia

**Keywords:** anticancer, ellagitannins, metabolites, polyphenols, urolithins

## Abstract

Cancer as a disease continues to ravage the world population without regard to sex, age, and race. Due to the growing number of cases worldwide, cancer exerts a significant negative impact on global health and the economy. Interestingly, chemotherapy has been used over the years as a therapeutic intervention against cancer. However, high cost, resistance, and toxic by-effects to treatment have overshadowed some of its benefits. In recent times, efforts have been ongoing in searching for anticancer therapeutics of plant origin, focusing on polyphenols. Urolithins are secondary polyphenol metabolites derived from the gut microbial action on ellagitannins and ellagic acid-rich foods such as pomegranate, berries, and nuts. Urolithins are emerging as a new class of anticancer compounds that can mediate their cancer-preventive activities through cell cycle arrest, aromatase inhibition, induction of apoptosis, tumor suppression, promotion of autophagy, and senescence, transcriptional regulation of oncogenes, and growth factor receptors. In this review, we discussed the growing shreds of evidence supporting these secondary phenolic metabolites' anticancer properties. Furthermore, we have pointed out some of the future directions needed to establish urolithins as anticancer agents.

## Introduction

Cancer is a disease that is one of the most challenging public health concerns of all time and has become a threat to the well-being of the individual population as its morbidity and mortality rates continue to increase (1, 2). Sitting at the edge of being a leading cause of non-communicable deaths globally (just next to cardiovascular disease), the number of new cases and deaths due to cancer has been projected to rise due to increased population, age, and lifestyle changes that serve as risk factors for cancer (3). In 2018, it was estimated that 18.1 million people were living with cancer, and the number of deaths arising from it was put at 9.6 million (4, 5).

Despite the increasing public awareness and advances in diagnosis and treatment regimens, there have been drawbacks arisen from drug resistance and increment in overall treatment cost in addition to unwanted side effects from anticancer drugs (2). Hence, efforts have been ongoing to look for a safer, cheaper, and more responsive chemoprevention strategy for cancer treatment. Such strategy involves the use of phytochemicals of natural origin to delay the onset of cancer, prevent or cure it (6, 7); culminating in the increased interest in research involving the search for anticancer agents in medicinal herbs and other plant materials (1). These phytochemicals have been shown to possess anticancer activities in animal models. They exert their biological effects through apoptosis induction, suppression of inflammatory reactions, and mitotic inhibition at different cancer development stages (8).

## Polyphenols as Anticancer Agents

Polyphenols are phytochemicals in foods, and they are present in abundant levels in fruits and vegetables. They exist in simple forms, such as in flavonoids or conjugated with sugars or organic acid. However, only a small fraction of the polyphenols are absorbed in the small intestine, where they are later metabolized by the gut microbiota. Thus, their biological function depends on the amount eaten in the form of food and by the tissue-derived metabolites or those obtained through the gut-derived microbial action (9). An increasing body of knowledge has supported the fact that polyphenols' consumption from time to time reduces the susceptibility to colorectal cancer (10), prostate cancer (11), cervical cancer (12), and breast cancer (13, 14). The anticarcinogenic effect of polyphenols involves several mechanisms. It includes but not limited to regulation of cellular signaling involving cancer cells, cell proliferation inhibition, induction of apoptosis, modulation of metastasis and angiogenesis, autophagy, epigenetic modification, and influence on the rate of change in the progression of the cell cycle (2, 12, 15–19).

## Ellagitannins and Ellagic Acid as Sources of Urolithins

The search for efficient, cost-effective, and harmless anticancer agents of natural origin is currently ongoing. One of such compounds that offer a promising prospect is ellagitannins (20). Ellagitannins (ETs) are hexahydroxydiphenoic (HHDP) acid esters having a complex chemical structure with a D-glucose carbohydrate moiety (21). They are either monomeric, i.e., having one glucose core with a different attachment of HHDP groups such as the punicalagin or polymeric ellagitannins, formed as a result of the polymerization of two or more monomeric ET units such as sanguiin H-6. As hydrolyzable tannins, they undergo hydrolysis producing HHDP, which is then spontaneously converted into ellagic acid (21). Furthermore, the ellagitannins form complexes with proteins and polysaccharides, and this forms part of the defense system used by plants for the protection against animal and bacterial attacks (22). They are found in many fruits, beverages, and nuts such as blackberries, strawberries, pomegranates, black teas, almonds, walnuts, and pecans. However, their chemical and biological reactivity depends on their chemical structure (23). This structural complexity and the ellagitannins' susceptibility to hydrolysis are critical for their potential health benefit (24). The ellagitannins possess various biological activities, including anti-inflammatory, antioxidant, antimicrobial, and anticancer activities (21, 25, 26).

Ellagic acid is a natural phenolic, double lactone ring compound of hexahydroxydiphenic acid (27). It is found in plants as a glucoside or as part of ellagitannins (15). A large amount of ellagic acid can be found in raspberries, strawberries, pomegranate, pecans, cranberries, and walnuts (15, 27, 28). Ellagic acid has been reported as having chemopreventive, radical scavenging, and antiviral properties (29). Other activities include anti-atherosclerosis, anti-hypertensive, anti-bacterial, and anti-inflammatory activities (25, 30). Its antitumor mechanism of action has been linked to its pro-apoptotic and antiproliferative properties (31, 32). For example, ellagic acid can act as an inhibitor of angiogenesis, extracellular matrix (ECM) invasion, and inhibitor of cell migration (32).

## Urolithins as Anticancer Agents

**Figure 1:**
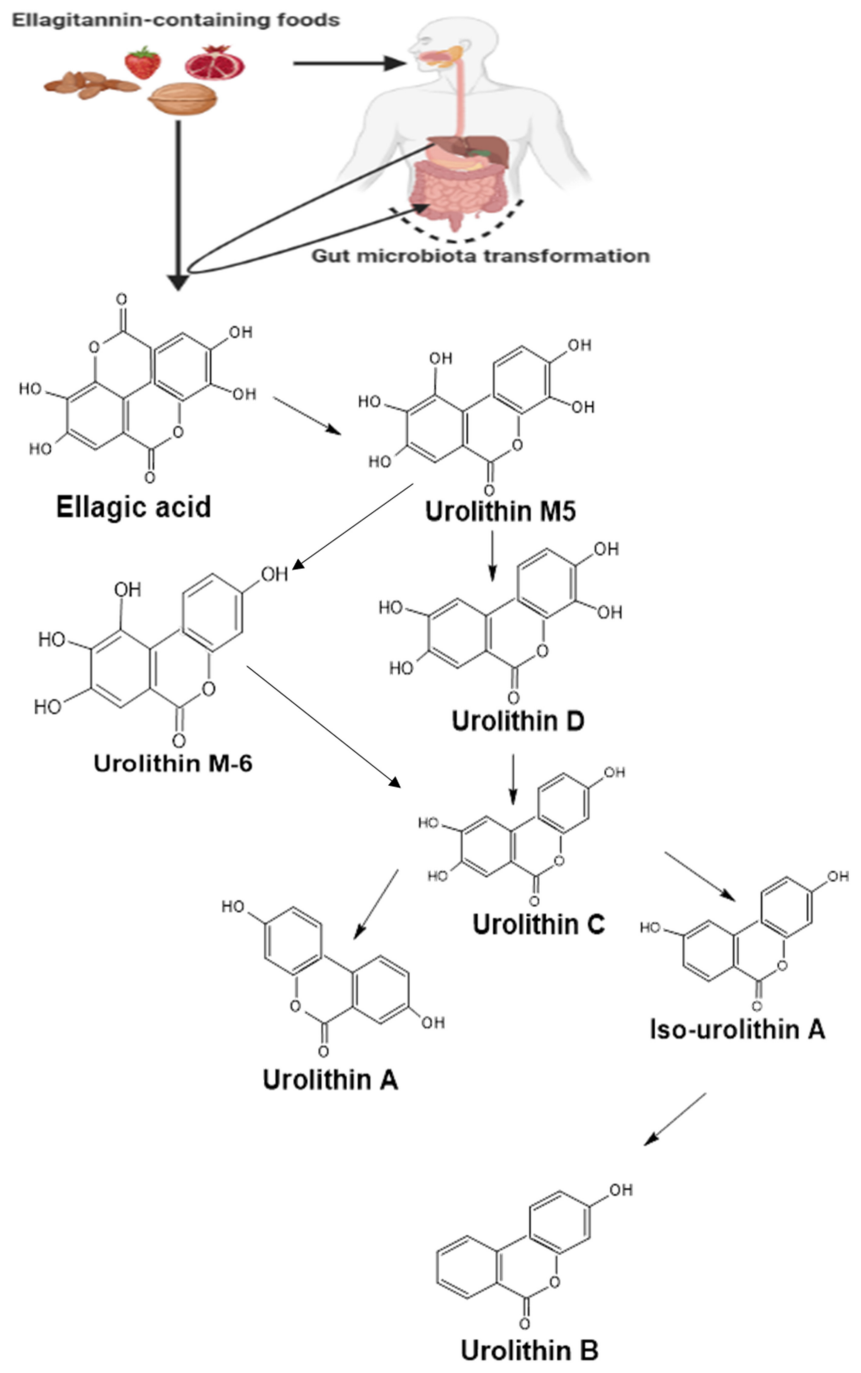
A summarized pathway for the formation of urolithins from ellagitannin and ellagic acid in the gut. Following the ingestion of food containing ellagitannins, they are hydrolyzed in the stomach to yield ellagic acid. The ellagic acid then undergoes series of transformations by the gut microbiota forming different urolithin molecules. Created with ChemSketch and BioRender.com.

Urolithins are the dibenzopyran-6-one secondary metabolites obtained from ellagic acid (EA) or ellagitannin (ET) rich foods such as pomegranate, berries, nuts, and oaks following gut microbial action (33). Following the ingestion of ET-rich foods, ET undergoes hydrolysis in the gut to EA. This is then subsequently metabolized through decarboxylation followed by dehydroxylation reactions by the gut microbiota to form different urolithin intermediates (Figure 1) such as urolithin D (Uro-D), urolithin C (Uro-C), urolithin A (Uro-A), and urolithin B (Uro-B) (34, 35); with Uro-A and Uro-B serving as the major metabolites present in the gut (36) and Uro-A as the most biologically active as compared to the rest of the metabolites (37). The produced urolithins are more lipophilic than the EA, and this has been suggested as a factor responsible for the greater urolithins absorption rate as compared to EA (38).

The metabolic breakdown of ellagitannins and ellagic acid into urolithins depends on the person's gut microbiota composition. Individuals metabolizing ET and EA into urolithins are categorized into three groups or phenotypes called metabotypes. While those that produce Uro-A are classified as metabotype A, the producers of Uro-A, IsoUro-A, and Uro-B are classified under metabotype B. Individuals who do not produce any of the urolithins or produce it at a none detectable level are grouped under metabotype 0 (35, 38–40).

**Figure 2:**
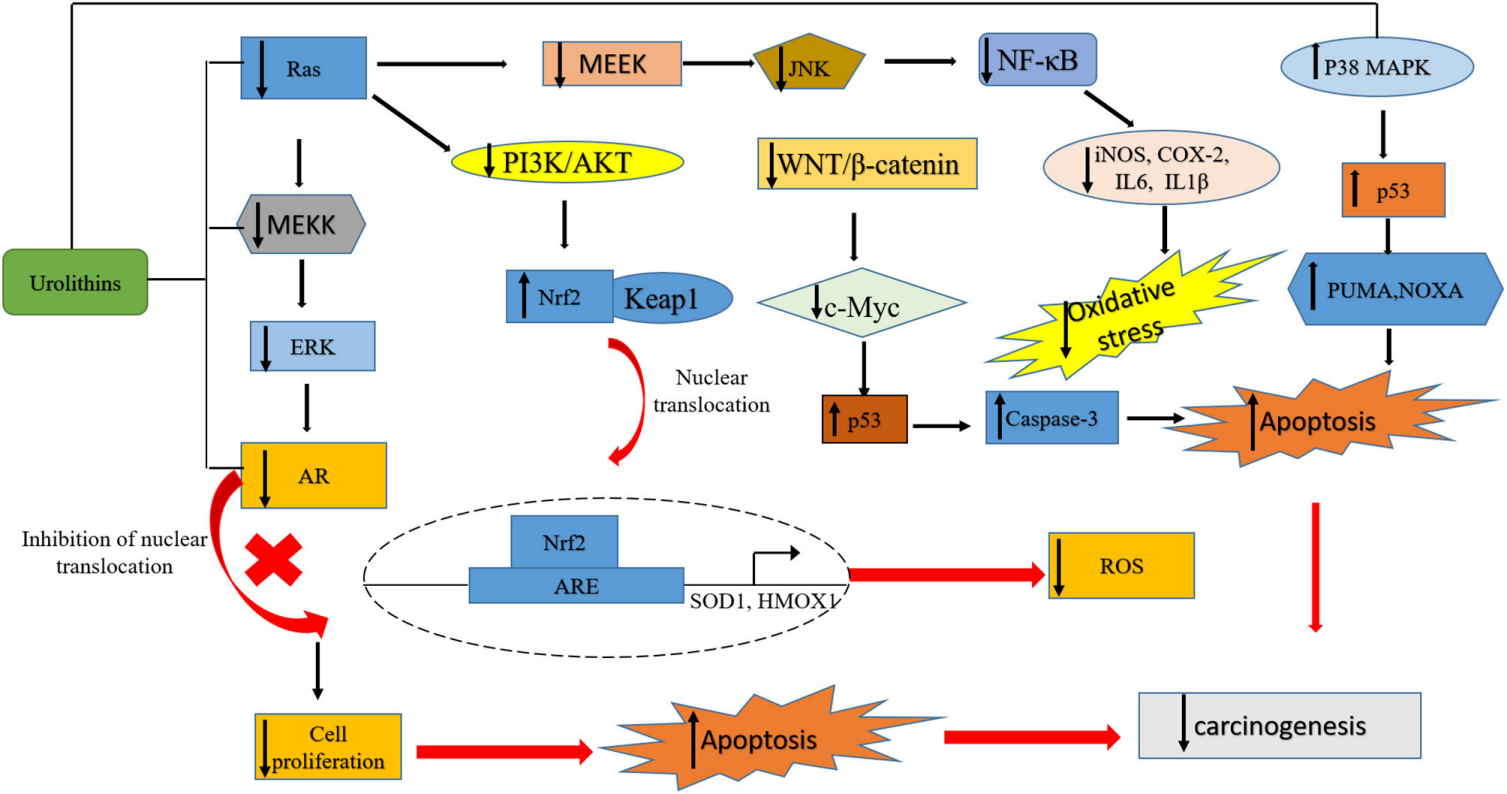
A summary of pathways targeted by urolithins in mediating their anticancer activity. The urolithins mechanism of action involves decreased oxidative stress, cell cycle arrest, inhibition of survival pathways, induction of apoptosis, tumor suppression, promotion of autophagy and senescence, transcriptional regulation of oncogenes, inhibition of growth factor receptors, and upregulation of tumor suppressor genes.

Urolithins have been reported to exhibit good bioavailability compared to ET and EA, and they are detected in concentrations at a micromolar range in plasma and urine samples (41). In humans, urolithins have been detected at a significant concentration in different tissues such as breast (42), colon (41) and prostate (43). The bioavailability of urolithins has been recently reviewed here (44). Following their absorption, urolithins can reach different parts of the body where they mediate various biological functions such as anti-obesity (45), antimicrobial, anti-inflammatory, anticancer (36, 46). Their anticancer effects (Table 1) are thought to be achieved through the regulation of expression of oncogenes, genes that mediate cell cycle, tumor suppressors, and growth factor receptors (72–74) (Figure 2).

**Table 1:** *In vitro* mechanism of action of urolithins.

**Metabolite**	**Cell line**	**Mechanism of action**	**References**
Uro-A	HepG2.2.15	Inhibition of cell proliferation and invasion	(47)
Uro-A and Uro-B	HT-29 and Caco-2	Cycle arrest at the G_2_/M and induction of apoptosis	(48)
Uro A and Uro-B	Caco-2	Modulation of phase I and phase II enzymes activities	(49)
Uro-A	PC-3 and C4-2B	AR/pAKT signaling inhibition	(50)
IsoUro-A and Uro-A	Caco-2 and CCD18-Co	Cell cycle arrest at S, G_2_/M phase, and apoptosis induction	(37)
Uro-A, Uro-B, Uro-C, Uro-D, and IsoUro-A	Caco-2, HT-29, and SW480, CCD18	Cell cycle arrest at the S phase in addition to G_2_/M for Uro-A and IsoUro-A. CDKN1A induction.	(51)
Uro-A, Uro-B, Uro-C, and Uro-D	Caco-2, SW480, and HT-29	Cell cycle arrest at the S and G_2_/M phases	(52)
Uro-A	RAW264	Suppressed NF-κB, AP-1, and inhibited pAKT and pJNK	(53)
Uro-A, Uro-B, M-Uro-A, M-Uro-B, and Uro-B sulfate	MCF-7aro	Inhibit aromatase activity	(54)
Uro-A and Uro-B	MCF-7	Estrogenic and antiestrogenic	(55)
Uro-A, Uro-B, and Uro-C	UMUC3	Cell cycle arrest at S phase by Uro B and G_2_/M for Uro-A in addition to inhibition of apoptosis, pAkt, and pERK	(20)
Uro-A	HEK 293T	Inhibition of the wnt signaling pathway	(56)
Uro-A	LNCaP64	Increase in Cell at G_1_ phase, apoptosis induction, caspase 3, and 7 activations, p21 upregulation	(18)
Uro-A, Uro-B, and Uro-C	LNCaP and DU-145	Apoptosis induction and PSA secretion modulation	(57)
Uro-A	HCT116	Inhibition of cell growth, cell cycle arrest at the G_2_/M phase, induction of p53 stabilization and upregulation of p21 and TIGAR gene expressions	(58)
Uro-A	Caco-2, SW-480, and HT-29	Inhibition of cell proliferation, cell cycle arrest at G_2_/M phase in Caco-2 and SW480 cells, in addition to S phase arrest in all cell lines when co-treated with 5-FU and 5′DFUR, apoptosis induction, caspase 8 and 9 activation	(59)
Uro-A, Uro-B, and Uro-C	LNCaP	Inhibit cell proliferation, inhibit arginase activity, and reduced PSA secretion except for Uro B	(60)
Uro-A and Uro-B	LNCaP	Decreased AR expression, reduced PSA levels, and induced apoptosis	(17)
Uro-A	HepG2	Suppression of β-catenin signaling, upregulation of p53, p38-MAPK, and caspase-3 expression, reduced intracellular ROS level, elevated intracellular SOD, and GSH-Px activity.	(61)
Uro-A	HEC1A and Ishikawa	Cell cycle arrest at the G_2_/M phase modulates the expression of GRIP1 and ERα.	(62)
Uro-A	sw620	Cell cycle arrest at G_2_/M phase, autophagy and apoptosis induction	(63)
mUA	DU145	Induction of apoptosis and mitochondrial depolarization decreased the expression of miR-21, pAkt, and elevated PTEN expression	(64)
Uro-A	HSFs	Cell cycle arrest at G_2_/M phase, reduced intracellular ROS level, increased expression of type I collagen, and a decrease in the expression of MMP-1.	(65)
Uro-A, Uro-B, and 8-OMeUro-A	T24	Increase expression of p38 MAPK, decrease expression of MEKK1 and P-c-Jun, induced apoptosis, decreased levels of intracellular ROS, MDA, and increased intracellular SOD	(66)
Uro-D	PC3	Inhibition of EphA2 phosphorylation	(67)
Uro-A, UM-A, UM-B, and IsoUro-A	HCT-116, Caco-2, HT-29, and CCD18-Co (normal cell)	Inhibition of colony formation in HCT-116, Caco-2 cells, and HT-29 by only Uro-A. cell cycle arrest at G_2_/M phase in HCT-116, Caco-2 cells, induction of cellular senescence by Uro-A and UM-A in HCT-116	(68)
Uro-A, Uro-B, IsoUro-A, Uro-A 3-glur, IsoUro-A 3-glur, Uro-A 3-sulf, Uro-B 3-glur, and Uro-B 3-sulf	MCF-7 and MDA-MB-231	Antiproliferative, estrogenic activity, and antiestrogenic activity	(69)
Uro-A, Uro-B, Uro-C, and Uro-D	HT-29 cells	Dose- and time-dependent decrease in cell proliferation, decrease clonogenic size and number, cycle arrest at the G_0_/G_1_ and G_2_/M (Uro-A and Uro-B), induction of apoptosis.	(70)
MPhA and MPhB	Caco-2	Inhibition of the number and size of colonospheres and inhibition of ALDH activity (MPhA)	(71)

*AP, Androgen Receptor; CDKN1A, Cyclin-Dependent Kinase Inhibitor 1A; NF-κB, Nuclear Factor-κB; AP-1, Activator Protein-1; ERα, Estrogen Receptor-α; mUA, Methylated Urolithin A; MMP-1, Matrix Metalloproteinase-1; HSFs, Human Skin Fibroblasts, ROS, Reactive Oxygen Species; SOD, Superoxide Dismutase; GSH-Px, Glutathione Peroxidase; PTEN, Phosphatase and Tensin Homolog, GREB1, Growth Regulation By Estrogen in Breast Cancer 1; MDA, Malondialdehyde; 5′DFUR, 5-deoxy-5-fluorouridine; 5-FU, 5-fluorouracil; UM-A, urolithin metabotype A; MPhA, mixed metabolites mimicking metabotype A; MPhB, mixed metabolites mimicking metabotype B*.

This review presents recent evidence of urolithins acting as anticancer agents at the preclinical stages in different cancer types. We started by providing background on these cancer types, followed by urolithins acting as anti-cancer agents in these cancer types.

## Urolithins on Prostate Cancer

Prostate cancer is one of the most common forms of cancer affecting men and responsible for 20% of all new cancer cases (75). Its incidence rate is increasing and occurs majorly in western countries due to environmental factors and lifestyle changes (76). The serum value of Prostate-Specific Antigen (PSA) is used as a biomarker to screen and diagnose prostate cancer. However, this method often poses a serious concern as prostate cancer is often over-diagnosed and leads to over-treatment (75). Besides, PSA is involved in the invasion, metastasis, and promotion of tumors. Its expression is under androgen regulation through the androgen receptor (17). One of the approaches employed in treating prostate cancer is androgen deprivation therapy, which involves either decreasing the circulating androgen level or inhibiting its interaction with its receptor (17, 57).

Urolithins mediate their chemopreventive potentials in prostate cancer in a dose-dependent manner, which is associated with the induction of apoptosis, upregulation of p21, and cell cycle arrest (17, 18, 57, 60, 77). In LNCap prostate cancer cell lines, treatment of these cell lines with Uro-A (40 μM) and B (40 μM) induced apoptosis and significantly inhibited prostate cancer cells' growth as evidenced from the cell cycle arrest at S and G_2_/M phases. The growth inhibition is associated with a time-dependent decrease in PSA and androgen receptors' mRNA level and protein expression. This decrease also resulted in the reduced interaction between the AR and its response element (RE), leading to PSA transcription inhibition (17, 18). Urolithin C at a lower concentration (IC_50_ = 35.2 ± 3.7 μM) showed a similar effect in LNCap prostate cancer cells (60).

The antiproliferative potential of the methylated form of Uro-A (mUA) has also been investigated in a prostate cancer cell line. Treatment of DU145 prostate cancer cell line with mUA (IC_50_ 44.3 ± 2.9 μM, 48 h) resulted in a dose-dependent inhibition of cell proliferation, induction of apoptosis with the activation of caspase pathway, decrease expression in Bcl-2/Bax ratio, and the depolarization of the mitochondria. Besides, the apoptotic induction, which is dependent on the expression levels of PTEN and Pdcd4, has been found to involve the downregulation in the expression of miR-21 and PI3K/Akt/β-catenin pathway inhibition (64). This chemopreventive property of mUA appears to be of significant importance since miR-21 is implicated in prostate cancer and other cancer types, and its overexpression is often associated with cancer cell invasion and metastasis (78, 79). *In vivo*, intraperitoneal injection of mUA (80 mg/kg) for 4 weeks significantly decreased tumor volume in DU145 xenograft mice. The decreased tumor volume was associated with decreased miR-21 expression and increased protein expression of PTEN (64), confirming the observed *in vitro* effect.

Urolithin A's chemopreventive effects have been tested on androgen receptor-negative prostate cancer cell lines such as PC-3 and androgen receptor-positive prostate cancer cell lines such as C4-2B. Dahiya et al. (50) reported that the Uro-A (35 μM) treatment of prostate cancer cell lines, PC-3 and C4-2B, resulted in cell growth arrest and induction of apoptosis with the activation of caspase-3 and PARP. This effect involves the inhibition of androgen receptor signaling. They reported that Uro-A at this concentration exerted this apoptotic effect in about 40% and 11% of C4-2B and PC-3 cell lines, respectively. *In vivo*, non-toxic oral administration of Uro-A (50 mg/kg) to mice inhibited C4-2B xenograft growth, which was associated with the downregulation of the androgen receptor, and pAKT signaling pathways. This Uro-A inhibitory activity is very much relevant within the context of castration-resistant prostate cancer (CRPC) since it has been shown that between 15 and 20% of patients developed resistance to androgen ablation therapy (a standard treatment option for prostate cancer) and progressed into CRPC due to the activation of other prosurvival pathways such as PI3K/AKT signaling (80).

A similar study explored the use of urolithins in combination therapy for cancer treatment. The authors studied the interactions between urolithins and bicalutamide (a clinically used non-steroidal antiandrogen) on LNCaP (androgen-dependent) and DU-145 (androgen-independent) cell lines. At an increasing concentration of (10–40 μM), urolithins A, B, and C individually inhibited prostate cancer cell proliferation. Uro-C's antiproliferation effect was more effective on DU-145 cell lines than Uro A and B, which were more effective on LNCaP cells. In combination with bicalutamide (10 μM), both Uro-A and B had similar addictive effects on LNCaP cells' inhibition. Uro-C antagonized the effect of bicalutamide (57). This result showed the potential use of Uro-A and Uro-B in combination therapy to improve prostate cancer treatment.

The Eph-ephrin system consists of a network of proteins that take part in many pathophysiological processes (81). This system is essential in controlling various developmental processes as well as in maintaining adult tissue homeostasis. Its abnormal function has been implicated in various diseases, including cancer. Hence, the Eph receptors are potential treatment targets for cancer (82). In mammals, including humans, nine EphA and five EphB receptors are present (83). Previous studies on the activation of EphA2 in prostate cancer cell showed the involvement of this receptor in cell adhesion, metastasis, and invasion (84). Uro-D's potential to interfere with the Eph signaling pathway has been tested on PC3 human prostate cell line. Using an ELISA binding assay, the authors showed that UroD (50 μM) exerted a selective EphA ephrin-A inhibition with an IC_50_ range of 0.14–4 μM and exhibited a competitive and reversible inhibition on EphA receptors with an inhibition constant, Ki of 312 nM on EphA2 receptor. Uro-D (IC_50_ 0.7 μM) also dose-dependently blocked the ephrin-A1-induced phosphorylation of EphA2 but without any cytotoxic and antiproliferative activity on PC3 cells, showing that UroD is an inhibitor of protein-protein interaction of the EphA system (67).

## Breast Cancer

Breast cancer is the leading cause of death in women <60 years of age and ranked second to all deaths arising from cancer (85). The real cause of breast cancer is still largely unknown (86). About 1 in 8 women have breast cancer, and this rate is rising globally despite concerted efforts to prevent it. The current treatment options include chemotherapy, hormone therapy, radiotherapy, and breast tissue removal (85, 87). Some breast cancer cells depend on estrogen for proliferation, which is a hormone that stimulates the increase in the rate of breast cancer cell proliferation. However, estrogen depends on the enzyme aromatase for its formation from androgen. Hence, a potential strategy to prevent breast cancer cells' growth would be through the targeting of this enzyme for inhibition of its activity so that the synthesis of estrogen can be halted.

Uro-A and Uro-B have been shown to possess antiproliferative, dose-dependent estrogenic, antiestrogenic, and anti-aromatase activities in breast cancer cell lines (54, 55). The urolithins' cancer-preventive potentials on hormone-dependent cancer cell proliferation have been investigated in MCF-7aro cells (cells overexpressing the enzyme aromatase). In addition to their aromatase inhibitory activities, Uro-A, Uro-B, methylated Uro-B, and Uro-B sulfate at a concentration of (47 μM) inhibited both the testosterone-induced proliferation and estrogen-induced proliferation of MCF-7aro cells (54), thus suggesting an ER signaling antagonist potentials for the metabolites. As noted by Larrosa et al. (55), both Uro-A and Uro-B showed improved antiestrogenic activity (quantified by their potentials to inhibit the proliferation of MCF-7 in the presence of 1 pM 17β-estradiol) in MCF-7 breast cancer cell line than most phytoestrogens with 0.4 and 0.75 μM IC_50_ values for urolithin A binding assays with ERα and ERβ and 20 and 11 μM IC_50_ values for Uro-B binding assays with ERα and ERβ, respectively.

The anticancer activity, the estrogenic and the antiestrogenic activities of urolithins aglycones (Uro-A, Uro-B, IsoUro-A) and their phase II metabolites (Uro-A 3-glur, IsoUro-A 3-glur, Uro-A 3-sulf, Uro-B 3-glur, Uro-B 3-sulf) have been compared in breast cancer cell lines. Using MDA-MB (estrogen negative cell; cells not expressing the estrogen receptor) and MCF-7 cells (estrogen-positive cells, cells expressing the estrogen receptor), the authors showed that at a metabolite concentration of 50 μM; IsoUro-A, Uro-A, and Uro-B exerted a decreasing antiproliferative potential (60, 35, and 25%, respectively), in MDA-MB cells. These antiproliferative activities were also confirmed for Uro-A (50 μM) and IsoUro-A (50 μM) on MCF-7 cell lines. The phase II metabolites had no growth-inhibitory potentials on MCF-7 cells. However, Uro-A 3-glur, IsoUro-A 3-glur, and Uro-B 3-glurs revealed a significant growth inhibition against MDA-MB cells (69). Furthermore, using a sensitive E-screen cell proliferation assay, the author also studied the estrogenic (defined as the capacity to induce proliferation of human ER-positive breast MCF-7) and antiestrogenic activities (defined as the capacity to prevent or diminish their proliferation in the presence of 17β-estradiol) of the metabolites. According to the data, all the aglycones (50 or 10 μM) tested exerted both estrogenic and antiestrogenic activity to a significant level. No estrogenic activity was recorded for the phase II metabolites at the concentration tested (50 or 10 μM). However, only the glucuronides of Uro-A and IsoUro-A (Uro-A 3-glur, Uro-A 8-glur, and IsoUro 3-glur) at the highest assay concentration (50 μM) exerted a weak preventive potential against 17β-estradiol-induced cell proliferation (69). These data signify that phase-II metabolism lowers the antiproliferative, estrogenic, and antiestrogenic activities of urolithin aglycones on breast cancer cells and agrees with a similar study on colon cancer reporting lower antiproliferative activities of the phase II metabolite (52).

## Uterine Cancer

Endometrial cancer ranked fourth in all cancer cases affecting women and in the United States, accounting for about 7% of all newly diagnosed cancers (88). With the increase in obesity cases globally, endometrial cancer incidence is also rising both in developed and lower economic countries, with a doubling rate seen in the last two decades (89). Indeed, most patients who have endometrial cancer are also obese (62). The increase in the adipocyte number and size in obese individuals contributes to increased circulating estrogen levels and thereby responsible for stimulating endometrial cancer proliferation (62). The treatment for endometrial cancer involves hysterectomy, bilateral salpingo-oophorectomy, and pelvic node dissection (90).

Apart from acting as an agonist for the estrogen receptor and mediating the estrogen receptor expression, Uro-A (10 μM) inhibited the proliferation of endometrial cancer cells at the G_2_/M phase in a time and dose-dependent manner. Besides, it upregulated the expression of key regulators of the G_2_/M phase such as cyclin-B1, cyclin-E2, p21, phospho (p)-CDC2 (on Tyr15), Myt1, and CDC25B proteins. Uro-A chemopreventive activity in endometrial cancer is through its action as an estrogen agonist, mediated through an ERα-dependent mechanism. Moreover, Uro-A modulated ER-mediated gene expression such as PGR, pS2, and GREB through its binding to the ERE. Uro B (10 μM) also inhibited endometrial cancer proliferation (62). The ability of cancer cells to migrate to other cells and invade other tissues requires actin cytoskeleton reorganization, which is controlled by the duo of ras-related C3 botulinum toxin substrate 1 (Rac1) and p21 protein-activated kinase 1 (PAK1) (91). Rac1 is a member of the Rho GTPases family and functions as a central regulator of the actin cell cytoskeleton (92). It is overexpressed in many cancers, and a loss of its activity has been found to lead to tumor growth suppression (93, 94). PAK1, on the other hand, is a member of the serine/threonine family of protein kinases, which plays an essential role in cell proliferation, cell survival, and cytoskeletal dynamics, and it is highly overexpressed in human tumors (95). Treatment of endometrial cancer (Ishikawa cells) with Uro-A (20 μM) significantly decreased the activity and mRNA levels of Rac1 and PAK1, and this resulted in actin depolymerization, which is associated with a decreased cancer cell proliferation and migration (91). This study points to the potential preventive role of Uro-A in cancer metastasis.

## Hepatocellular Carcinoma

Hepatocellular carcinoma (HCC) ranked sixth amongst all cancer types and second amongst cancer-causing death. In 2012, about 780,000 hepatocellular carcinoma cases were reportedly diagnosed, accounting for about 750,000 deaths. HCC arises from damage to the liver cells due to inflammation leading to liver cell necrosis and fibrosis (96, 97). Certain conditions predispose one to HCC, including chronic infections from hepatitis B and C viruses, non-alcoholic steatohepatitis, liver cirrhosis, and metabolic diseases such as diabetes and obesity (97, 98).

In HCC caused by infection arising from HBV, let-7a (lethal-7), a member of the let family of miRNA, is an essential regulator of differentiation and functions as a tumor suppressor. It is often deleted in HCC and most cancer, and its aberrant expression and regulation are linked to cancer progression and poor patient prognosis (99). Let-7a is negatively regulated by Lin28a, an RNA binding protein that functions by recruiting Zcchc11 and caused the degradation of pre-let-7, thereby preventing its processing into functional let-7a miRNA (47). According to Qiu et al. (47), Uro- A (1–120 μM) exerts its cancer-preventive effect in HCC in a dose-dependent manner with the induction of cytotoxicity in HepG2.2.15 cell lines and suppression of tumor cell invasion, which was associated with the inhibition of K-ras/HMGA2 signaling. Furthermore, Uro-A treatment resulted in the upregulation of let-7a and repression in the protein expression of Lin28a, Zcchc11, and Sp-1, a transcriptional factor overexpressed in most cancer and a target for Lin28a activity.

The cancer-preventive potentials of Uro-A and Uro-B on hepatocellular carcinoma have been reported. *In vitro*, Uro-A in a dose and time-dependent manner inhibited the growth of HepG2 cells. This inhibitory effect was associated with increased cell cycle protein expression and apoptosis regulators such as the p53 and p38-MAPK and decreased c-Jun phosphorylation (61). Although the authors of this study reported an IC_50_ = 137 ± 8.2 μM for Uro-A growth inhibition on HepG2 cells, this seems to be the highest concentration ever to be reported for Uro-A anticancer activity *in vitro* and even more than the previously reported plasma concentration (0.024–35 μM) of Uro-A glucuronide (38). Extra caution must, therefore, be taken in extrapolating *in vitro* effect into what happens *in vivo*. Uro-B (15 μM), on the other hand, inhibited the proliferation of HCC and induced a significant cell cycle arrest at the G_0_/G_1_ phase in HepG2 cell and at the S phase in Bel7402 cells. Uro-B treatment also induced apoptosis, which is evident from the decreased protein expression of Bcl-2. *In vivo*, Uro-B (40 mg/kg) suppressed tumor growth in a xenograft mice model (100). Uro-B's antiproliferative potential in both *in vivo* and *in vitro* is associated with an increase in phosphorylation of β-catenin, blocking its translocation from the nucleus to the cytoplasm and resulted in the inactivation of Wnt/β-catenin signaling (61, 100).

## Colon Cancer

Colorectal cancer is a leading cause of death globally, affecting both genders in equal proportion. It is ranked third and fourth in terms of cancer's commonality and cause of death, respectively (101, 102). It slowly begins as a polyp in the interior lining of the rectal area of the colon. If left untreated, it metamorphoses into a cancer cell with the ability to be metastasized to other locations in the body. The consumption of high-calorie food, such as animal fat, can predispose one to colon cancer (103).

In colorectal cancer cell lines, urolithins exert their anticancer activity mostly through the promotion of apoptosis and cell cycle arrest (37, 48, 51, 52). In HT-29 colon cancer cell line, Uro-A (30 μg ml^−1^) and Uro-B (30 μg ml^−1^) mediate their antitumor potentials through apoptosis induction by activating caspase 3. These metabolites caused the upregulation in the expression of p21 protein and G_2_/M phase arrest of the cell cycle (48). In Caco-2 colon cancer cell lines, Uro-A in addition to Iso Uro-A, and in a time and dose-dependent manner (50–100 μM, 24–48 h) caused cell cycle arrest at both the S and G_2_/M phases (37, 52) and the S phase by Uro-B, Uro-C, and Uro-D (52). Uro-A, Uro-C, and Uro-D also arrested cell cycle progression at the S-phase in SW480 and HT29 colon cancer cells (52). The anticancer potentials of urolithins might be due to the number of functional groups in their chemical structure, the effects of ionization on their stability, and the ionic charges in their microenvironment (51). These results indicate that the anticancer effects of the urolithins are cell-dependent.

Autophagy is one of the hallmarks of cancer. It is also a physiological response of the cell in which cellular organelles like the ribosomes and mitochondria are broken down in the lysosomes. The catabolite obtained from the breakdown product is recycled and used for other metabolic processes and as a source of energy for the cell (104). Autophagy plays a critical role in colon cancer progression (105). At an early stage, autophagy inhibits tumor invasion and metastasis while promoting metastasis and survival at a later stage (63). Uro-A (15 μM) and (30 μM) concentrations resulted in the induction of autophagy in SW620 colorectal cancer cell lines as well as apoptosis, respectively. Treatment of these cells with Uro-A dose-dependently led to a decrease in cell proliferation and delayed cell migration, which was associated with the reduction in the activity of matrix metalloproteinase-9 (MMP-9) (an endopeptidase involved in metastasis and invasion). Uro-A exposure decreased DNA synthesis and inhibited movement through the cell cycle (63).

The urolithins have the potentials to inhibit the glycosylation of proteins. Glycosylation is a post-translation modification that involves an enzyme-assisted addition of carbohydrate chain or glycans to proteins and lipids. Aberrant glycosylation is seen in major diseases, including cancer (106). One common type of glycosylation is the mucin-type O-glycosylation, such as those involving the glycosylation of the glycoprotein podoplanin (PDPN). Moreover, such glycosylation is initiated by one of the 20 members of the polypeptide N-acetyl-α-galactosaminyltransferases (107). Abnormal expression of the PDPN is associated with tumor cell migration and invasion (108). Therefore, inhibition of glycosylation or the expression of PDPN will serve as a potential strategy to prevent tumor cell progression. Uro-D (40 μM) inhibited mucin-type O-glycosylation in HCT116, SW480, and RKO colon cancer cells. The inhibited O-glycosylation is associated with decreased PDPN expression and resulted in colon tumor cell migration and invasion inhibition (109).

The urolithins' potentials in modulating the expression of phase I and phase II detoxifying enzymes have also been studied in both colon cancer cell lines and *in-situ* rat model (49). The Phase I and II enzymes are enzymes with critical roles in the metabolism of chemical carcinogens such as polycyclic aromatic hydrocarbons (PAHs) (110). The phase I enzymes such as the cytochrome P450 (CYP), are involved mainly in oxidation, reduction, and hydroxylation reactions (111). The phase II enzymes such as the UDP-glucuronosyltransferases, glutathione transferases, and sulfotransferase are involved in conjugation reactions: conjugation of phase I metabolite (112). Interestingly, the phase I and phase II enzymes function to ultimately convert the PAHs and other environmental toxicants into a more polar and water-soluble metabolite that is finally excreted in bile or urine (112). According to González-Sarrías et al. (49), both Uro-A and Uro-B at concentration achievable *in vivo* (40 μM) induced the expression and activity of CYP1A1 and UGT1A10. Urolithin B also significantly induced CYP1B1 and CYP27B1 expressions in Caco-2 cells (49). The CYP27B1 enzymes take part in the synthesis of 1,25-diOH vitamin D3, an active metabolite of vitamin D that has been previously reported to protect against colon tumors' growth (113, 114). Paradoxically, the CYP1B1 enzymes have been reported to be involved in the activation of procarcinogens, and high expression of these enzymes have been observed in different human cancers (115, 116). Therefore, induction of the expression CYP1B1 by Uro-B is not a desirable effect required in cancer therapy. Although the induction of CYP1A1 has been shown to offer more protections against oral carcinogens, the induction of the expression CYP1B1 by Uro-B would be critical in CYP1A1 deficient individuals exposed to the toxic environmental substance. For the *in situ* rat model, Uro-A and Uro-B were dissolved in either PBS or sunflower oil. The authors noted an induction of CYP1A1 only in the colon of rats incubated with Uro-A and Uro-B dissolved in PBS and not in sunflower oil (49). The *in situ* results points to a critical effect of the dissolving media in the activities of the urolithins. Another study also confirmed the potential inhibitory effects of several urolithins metabolites on CYP1. According to Kasimsetty et al. (70). Uro-A (IC_50_, 56.7 ± 2.6 μM), Uro-B (IC_50_, 58.6 ± 4.2 μM), and Uro-C (IC_50_, 74.8 ± 2.29 μM) exerted dose-dependent inhibition of TCDD-induced CYP1 enzymes on HT-29 cells. These metabolites, including Uro-D, induced a dose and time-dependent antiproliferative action on HT-29 cells with IC_50_ values in the range of 316–378 μM. These weak albeit antiproliferative potentials are specific to cancer cells only and are associated with apoptosis induction (70).

Urolithin A has been showed to exert a synergistic action with oxaliplatin on colon cancer cells. Oxaliplatin is a standard chemotherapeutic drug used for therapy against colon cancer. Urolithin A in a time and dose-dependent manner (39.2 μM, 48 h, and 19.6 μM, 72 h) inhibited the growth of HCT116 cells and halted cell cycle progression at the G_2_/M phase. The Uro- A growth inhibitory effect on HCT 116 cells is p53-dependent at a low dose and p53 independent at a high dose. Uro- A also showed p53-dependent synergistic action with oxaliplatin as evidenced from the reported combinatorial indices (CI) of <1 (58). A CI value <1 denotes synergism, values > 1 indicates antagonism and values = 1 denotes an addictive effect (117). These study data imply that urolithin could aid oxaliplatin chemotherapy against colon cancer. Furthermore, cancer cells rely on aerobic glycolysis for glucose metabolism. This metabolic reprogramming from oxidative phosphorylation to glycolysis has been suggested to promote tumor cell growth and malignancy (118) and recognized as an emerging hallmark of cancer (104). An increased aerobic lactic acid production *via* glycolysis is associated with drug resistance in LoVo colon carcinoma cells (119). Thus, an interruption of cellular bioenergetics in tumor cells can sensitize the cell to chemotherapy and inhibit tumor growth through energy depletion. Using extracellular flux analysis, Norden and Heiss (58), showed that Uro- A influenced cellular bioenergetics in HCT 116 cells in a p53-dependent manner through a reduction in glycolytic potential. This reduced glycolytic potential is associated with the induction of TP53-induced glycolytic regulatory phosphatase (TIGAR) in WT HCT116 cells. TIGAR is a negative regulator of glycolysis. Its overexpression leads to a decrease in cellular fructose-2,6-bisphosphate levels, resulting in the inhibition of glycolysis (120). Thus, this study points to another Uro-A antiploriferative potentials against cancer cells.

Uro-A's combinational therapy with 5-Fluorouracil (5-FU) and 5-deoxy-5-fluorouridine (5′DFUR) has been examined on colon cancer cell lines. The 5′DFUR is a pro-drug and also an intermediate of 5-FU. The co-treatment of 5-FU with Uro-A increased the sensitivity of 5-FU in Caco-2 (1.2 and 2.4-fold), SW480 (1.6 and 2.4-fold), and in HT-29 cells (1.3 and 1.7-fold) in the presence of 10 and 20 μM, 72 h of Uro-A, respectively. The same increased sensitivity was observed when Uro-A at a non-toxic concentration of 10 or 20 μM was cotreated with 5′DFUR in Caco-2 (1.3 and 1.6-fold) in SW480 (1.8 and 2.3-fold), and in HT-29 cells (1.1 and 1.7-fold). This increased sensitivity from the co-treatment with Uro-A resulted in the decrease in the IC_50_ values of 5-FU and 5′DFUR against the cancer cell lines (59). A previous study also reported an increased sensitivity from the co-treatment of Uro-A with oxaliplatin. However, in contrast to Uro-A synergistic effects with oxaliplatin (58), here, Uro-A showed additive effect with 5-FU and 5′DFUR. This addictive effect resulted in a greater cell cycle arrest at the S and G_2_/M phases, increased cyclin A and B1 levels, induction of apoptosis, and caspase 8 and 9 activation (59). These data suggest a potential combinational therapy of Uro-A with 5-FU or its metabolic intermediate; 5′DFUR as a new treatment option to enhance the antitumor effect of 5-FU.

Cellular senescence is the permanent/irreversible inhibition of cell proliferation which is ensured when cells are exposed to stress conditions. It has been suggested as an antitumor mechanism through which cancer cell growth can be inhibited since the cancer cells' ability to proceed through the cell cycle is halted (121). The molecular signatures of cells undergoing senescence include the increased activity of β-galactosidase and the upregulation of p21, p16 gene expressions (122). The urolithins' ability to promote senescence has previously been investigated in colon cancer cell lines. For example, long term exposure of Uro-A (10 μM) and urolithin metabotype A (UM-A) (10 μM), [a representavive mixture of urolithins that mimics *in vivo* UM-A] for 5 days induced senescences in HCT-116 cells, which was associated with the upregulation of p53 and induction of p21 expressions (68). These data demonstrated Uro-A and UM-A's potentials at a non-toxic dose to prevent cancer cell proliferation through senescence induction. Apart from senescence induction, these two metabolites and other relevant urolithin metabolites also exhibited various biological activities in different colon cancer cell lines. For example, Uro-A (10 μM), UM-A (10 μM), and urolithin metabotype B (UM-B) (10 μM), [a representavive mixture of urolithins that mimics *in vivo* UM-B] decreased colony formation, with the inhibition of cell cycle progression at the G_2_/M phase by Uro-A and UM-A and at the S phase by IsoUro-A, Uro-C, and UM-B in HCT-116 cells. Uro-A (1, 10 μM), Uro-C (1, 10 μM), UM-A (10 μM), IsoUro-A (10 μM), Uro-B (10 μM), and UM-B (10 μM) on the other hand showed significant anticlonogenic activities against Caco-2 cells and also significantly inhibited movement through the cell cycle at the G_2_/M phase with the exception for Uro-C which showed a non-significantly cell cycle arrest at the S and G_2_/M phases in Caco-2 cells (68).

Cancer stem cells (CSCs) are a subgroup of cancer cells with unique features synonymous to other stem cells, such as unending cell division, self-renewal, and ability to differentiate into other cell types. The CSCs are essential in colon cancer relapse and metastasis. They possess specific markers located on the cell surfaces such as CD44, CD133, and aldehyde dehydrogenase (ALDH) activity, among others (123). An elevated ALDH activity has been linked with chemoresistance in colon cancer cells (124). The potential inhibitory effects of mixed metabolites MPhA comprising 85% Uro-A, 10% Uro-C, and 5% of EA and MPhB comprising 30% Uro-A, 50% IsoUro-A, 10% Uro-B, 5% Uro-C, and 5% EA on CSCs colony formation and size have been examined. According to the authors, MPhA at a mixed concentration of 17 μM Uro-A + 2 μM Uro-C + 1 μM EA and MPhB at a mixed concentration of 6 μMUro- A + 10 μM IsoUro-A + 2 μM Uro-B + 1 μM Uro-C + 1 μM EA inhibited colonsphere formation by 30.5 ± 12.1% and 38.9 ± 4.4% on non-adherent Caco-2 cells, respectively. These mixtures also decreased the spheroid size by 8.5 ± 5.9% for MPhA and 15.0 ± 2.8% for MPhB in Caco-2 cells with concomitant decrease in ALDH activity seen only for MPhA (71). These data agreed with previous studies on Resveratrol (125) and Epigallocatechin gallate (126) inhibition of CSCs and thus showed the potential modulatory effect of MPhA mixtures on CSC associated-chemoresistance of cancer cells.

## Bladder Cancer

Bladder cancer sits at the 9th position in cancer types' commonality and is one of the common cancer in humans (127). It is a multifaceted disease linked to increased morbidity and mortality when left untreated (128). Diagnosis is usually conducted in individuals above 50 years of age and involves a medical history, medical test and imaging, tissue cytology, and cystoscopic examination (127). Cisplatin is often the first choice drug to treat bladder cancer, but it is usually associated with adverse side effects and drug resistance (129).

The UMUC3 bladder cancer cell lines are model cell lines associated with an abnormality in cell cycle checkpoint, a hallmark of cancer that results in increased genetic instability and uncontrollable cell division (130). The antiproliferative effects of Uro-A, B, and C have been tested on these cells. These metabolites have a reducing effect on cell viability, with Uro-A being the most active metabolites. Uro-A inhibits cell cycle arrest at the G_2_/M phase in a time-dependent manner (20). This checkpoint arrest could be due to Uro-A's ability to inactivate the cyclin B1/cdc2 kinase complex, known for its regulatory function on the G_2_/M transition (131). Bladder cancer, in addition to its reliance on ERK pathway activation, is also associated with a defect in the PI3K/Akt signaling pathway, which ensures that cancer cells continue to proliferate and escape apoptosis (132–134). Hence, inhibition of these pathways could serve as a treatment strategy for bladder cancer. Intriguingly, Uro-A (23.92 μM) decreased the phosphorylation state of p-Akt and ERK 1/2 in the UMUC3 cell line, suggesting that Uro-A could serve as a potential therapeutic agent for bladder cancer (20).

## Future Prospective

In recent years, different researches have been accelerated to explore the therapeutic intervention of diet in managing many diseases, including cancer. Pomegranates and nuts are rich in polyphenolic compounds and have been reported well as a safe and emerging molecule for preventing and managing various cancer types. Urolithins have been found as a critical anticancer component of ellagitannin-rich food sources. The *in vitro* cardiovascular, anticancer, anti-inflammatory, and anti-diabetics therapeutic potentials of urolithins, are well-reported with just a few *in vivo* studies. Therefore, further studies are needed regarding the anticancer activities of urolithin using *in vivo* models. The bioavailability of urolithins in different cancer types should be further explored. This is important to establish the concentration of urolithins which can reach different target tissues. The knowledge of this bioavailability will be essential in developing realistic *in vitro* studies with physiological concentration.

Furthermore, urolithins' solubility is also a challenge for drug delivery, and novel drug delivery systems need to be developed using nanotechnology. It would be beneficial in absorption and distribution within the cell and to potentiate the therapeutic effects. The sensitivity of cancer cells to the phase II metabolites of urolithins at the molecular level is also not explored fully. Moreover, more *in vivo* studies and more synergistic efficacy of urolithins with other anticancer drugs also need to be further explored. Additionally, the anticancer potentiality of these therapeutic molecules must be evaluated through best-designed human clinical trials. Thus, more research is needed to overcome the above challenges and establish urolithins as an alternative new broad-spectrum anticancer molecule.

## Conclusion

The anticancer activities of the polyphenolic metabolites urolithins are evolving topics in cancer biology and one that will open doors to the development of new therapy for the management and treatment of various cancer types. As summarized in this review, the ellagitannin and ellagic acid anticancer properties are mainly due to their gut-derived metabolites, the urolithins. Many of the anticancer activities attributed to urolithins involve cell cycle arrest and apoptosis induction. Other mechanisms include modulation of pathways associated with cell proliferation, cell survival, oxidative stress, detoxification, and the modulation of pathways involving hormonal actions (Figure 2 and Table 2). It is noteworthy that oral administration of chemically synthesized urolithin A has been recently found to be safe in humans (135). Also, the US Food and Drug Administration has previously given Uro A a favorable review in its generally safe (GRAS) notification program, and 1,000 mg/serving of urolithin A can be used as a functional food ingredient (136).

**Table 2:** Urolithins targeted genes and their pathways.

**Metabolite**	**Gene**	**Targeted pathway**	**References**
Uro-A	let-7a, Lin28a, Zcchc11, and Sp-1	K-ras and HMGA2	(47)
Uro-A and Uro-B	p21 and PARP	Extrinsic and intrinsic apoptotic pathways	(48)
Uro-A	AR, AKT, PSA an dGSK 3α-β	AR signaling and AKT signaling	(50)
Uro-A and Uro-B	CYP1A1, CYP1B1, CYP27B1, CYP3A5, UGT1A10, UGT1A6, UGT2B15, UGT2B28, SULT1A1, SULT1A2, SULT1A3, SULT2A1, and SULT1C1	Glucuronidation, sulfonation	(49)
Uro-A and Uro-B	FGFR2, EGFR, K-Ras, c-Myc, DUSP6, Fos, CCNB1, CCNB1IP1, MAP4K4, and CD44	MAPK, K-Ras signaling	(72)
Uro-A	iNOS, IκB-α, NF-κB (p65), c-Jun, Akt and JNK, p38	PI3-K/Akt, NF-κB and JNK/AP-1 signaling	(53)
Uro-A, Uro-B, and Uro-C	Akt, ERK, SAPK/JNK, and p38	PI3K/Akt and MAPK	(20)
Uro-A, Uro-B, and 8-OMe-Uro-A	p38-MAPK, MEKK1, and c-Jun	MAPK and MEKK1	(66)
Uro-A	CDKN1A	Cell cycle and apoptosis	(18)
Uro-A and Uro-B	AR and KLK3 (PSA)	Androgen metabolism	(17)
Uro-A	β-catenin, c-Myc, and Cyclin D1, IL6, IL1β, NF-κB, COX-2, iNOS, p53, Bax, PUMA, NOXA, and p38 MAPK	Apoptosis, inflammatory, MARK, JNK, and Wnt signaling	(61)
Uro-A	ERα, ERβ, PGR, pS2, GREB1, and GRIP1	Estrogen receptor signaling	(62)
mUA	Bcl-2, Mcl-1, Bax, Bad, miR-21, PTEN, Pdcd4, MMP-7, c-Myc and Cyclin D1, FOXO3a and Akt	Wnt signaling, apoptosis, and Akt	(64)
Uro-A	MMP-1, collagen-1, SOD1, NQO1, GCLC, and HMOX1	Nrf2/ARE pathway	(65)
Uro-A	p53, p21, and TIGAR	Cell cycle, p53 signaling, glycolysis	(58)
Uro-D	EphA2 and EGFR	Eph signaling	(67)
Uro-A and UM-A	p53, p21^Cip1/Waf1^	Cellular senescence	(68)

*iNO, inducible NO synthase; NFκB, Nuclear factor-kappa B; AKT, Protein kinase B; COX-2, Cyclooxygenase 2; CYP, Cytochrome P450; ERα, estrogen receptor-α; Mcl-1, Myeloid cell leukemia 1; SOD1, superoxide dismutase 1; NQO1, quinone 1; GCLC, glutamate-cysteine ligase catalytic subunit; HMOX1, heme oxygenase 1; mUA, Methylated urolithin A; GREB1, Growth regulation by estrogen in breast cancer 1; GRIP1, Glutamate receptor-interacting protein 1; PTEN, Phosphatase and tensin homolog; MMP-1, Matrix metalloproteinase-1; MMP-7, Matrix metalloproteinase-7; PUMA, p53 upregulated modulator of apoptosis; NOXA, NADPH oxidase activator 1; FOXO3, Forkhead Box O3; PGR, Progesterone receptor; KLK3, Kallikrein Related Peptidase 3; AR, Androgen Receptor; TIGAR, TP53-induced glycolytic regulatory phosphatase; EphA2, Erythropoietin-producing hepatocellular A2; EGFR, Epidermal growth factor receptor*.

The urolithins anticancer activities are comparable to other established polyphenols with anticancer potentials such as curcumin and resveratrol. For example, curcumin, one of the numerous phenolic pigments found in nature, is obtained from the plant *Curcuma longa L*. Its anticancer activities in numerous cancer types have been attributed to its potential to modulate cell differentiation, cell cycle arrest, and apoptosis (137). Curcumin causes the suppression of NF-κB (a transcription factor whose constitutive expression is implicated in many cancers), leading to a decrease in its target genes such as COX-2 and cyclin D1 and ultimately leading to apoptosis (4). Furthermore, curcumin inhibits cell growth and invasion through the downregulation of EGFR and MMP-2 genes' expression, respectively (6).

Similarly, resveratrol is a dietary polyphenol obtained from plants. Its ability to cause cell cycle arrest and induce apoptosis has been demonstrated in both *in vivo* and *in vitro* cancer models (138). Resveratrol inhibits metastasis in colon cancer cells by decreasing the expression of hypoxia-inducible factor-1α (HIF-1α) and MMP-9 (139). In prostate cancer, resveratrol has been found to attenuate cell proliferation and upregulate the induction of apoptosis by either decreasing the activation of MAPK or NF-κB induced inactivation (140). The mechanisms of action of curcumin and resveratrol are similar to what has been reported so far for the urolithins (Table 2).

However, as most of the urolithins' reported anticancer activities were conducted through *in vitro* studies, caution must be made to translate it into what happens *in vivo*. Nevertheless, the research on urolithins will be an interesting one in the coming days ahead.

## Author Contributions

SA-H, AA, MZ, and MK contributed to the manuscript's conception and development. AA was responsible for the scientific writing of the manuscript. SA-H, MZ, and MK contributed to the manuscript's review. SA-H was responsible for the source of funding. All authors contributed to the manuscript and approved the submitted version.

## Conflict of Interest

The authors declare that the research was conducted in the absence of any commercial or financial relationships that could be construed as a potential conflict of interest.
